# Commonly used anesthetics modify alcohol and (-)-trans-delta9-tetrahydrocannabinol in vivo effects on rat cerebral arterioles

**DOI:** 10.1186/s12871-023-02320-9

**Published:** 2023-12-12

**Authors:** Steven Mysiewicz, Brianne Hibl, Alex Dopico, Anna Bukiya

**Affiliations:** 1 Department of Pharmacology, Addiction Science and Toxicology, College of Medicine, The University of Tennessee Health Science Center, 71 S. Manassas, Memphis, TN 38103 USA; 2 Laboratory Animal Care Unit, The University of Tennessee Health Science Center, Memphis, TN 38103 USA

**Keywords:** Cerebral artery, Myogenic tone, Pial arteriole diameter, Ketamine, Xylazine, Isoflurane

## Abstract

**Background:**

Ethyl alcohol and cannabis are widely used recreational substances with distinct effects on the brain. These drugs increase accidental injuries requiring treatment under anesthesia. Moreover, alcohol and cannabis are often used in anesthetized rodents for biomedical research. Here, we compared the influence of commonly used forms of anesthesia, injectable ketamine/xylazine (KX) versus inhalant isoflurane, on alcohol- and (-)-trans-delta9-tetrahydrocannabinol (THC) effects on cerebral arteriole diameter *evaluated* in vivo.

**Methods:**

Studies were performed on male and female Sprague–Dawley rats subjected to intracarotid catheter placement for drug infusion, and cranial window surgery for monitoring pial arteriole diameter. Depth of anesthesia was monitored every 10–15 min by toe-pinch. Under KX, the number of toe-pinch responders was maximal after the first dose of anesthesia and diminished over time in both males and females. In contrast, the number of toe-pinch responders under isoflurane slowly raised over time, leading to increase in isoflurane percentage until deep anesthesia was re-established. Rectal temperature under KX remained stable in males while dropping in females. As expected for gaseous anesthesia, both males and females exhibited rectal temperature drops under isoflurane.

**Results:**

Infusion of 50 mM alcohol (ethanol, EtOH) into the cerebral circulation rendered robust constriction in males under KX anesthesia, this alcohol action being significantly smaller, but still present under isoflurane anesthesia. In females, EtOH did not cause measurable changes in pial arteriole diameter regardless of the anesthetic. These findings indicate a strong sex bias with regards to EtOH induced vasoconstriction.

Infusion of 42 nM THC in males and females under isoflurane tended to constrict cerebral arterioles in both males and females when compared to isovolumic infusion of THC vehicle (dimethyl sulfoxide in saline). Moreover, THC-driven changes in arteriole diameter significantly differed in magnitude depending on the anesthetic used.

Simultaneous administration of 50 mM alcohol and 42 nM THC to males constricted cerebral arterioles regardless of the anesthetic used. In females, constriction by the combined drugs was also observed, with limited influence by anesthetic presence.

**Conclusions:**

We demonstrate that two commonly used anesthetic formulations differentially influence the level of vasoconstriction caused by alcohol and THC actions in cerebral arterioles.

## Introduction

Alcohol (ethyl alcohol, ethanol, EtOH) and cannabinoids are among the most widely used psychoactive drugs in the world with an alarming growing trend toward their simultaneous consumption [1, 2]. Both have distinct effects in the brain and have been implicated in the progression of major cerebrovascular pathologies. Specifically, recreational episodic drinking with blood EtOH levels reaching 35–80 mM is associated with an increased risk for cerebrovascular ischemia [3], stroke and death from ischemic stroke [4]. Likewise, cannabinoids substantially increase risk for ischemic stroke [5, 6], and are recognized as a trigger for cerebrovascular malfunction in the absence of other risk factors [6, 7].

While neurons have been widely acknowledged as major targets of recreational drugs, the growing number of reports has been linking neuronal malfunction to alterations in cerebral perfusion [8, 9]. Owing to the strong similarities between human and rodent brain circulations [10], EtOH and cannabinoid actions on cerebral artery and arteriole diameter are often studied at laboratories using rodent models [11–13]. In a rat model that mimics the human cerebral artery from morphological and developmental standpoint [10], in vivo imaging and ex vivo studies detected EtOH-induced cerebrovascular constriction [13–15]. This observation resembles findings in the human brain where albeit some regional variability, decrease in cerebral perfusion following excessive EtOH consumption has been usually reported [16, 17]. With regards to cannabinoids, human and rodent data are less consistent. While (-)-trans-delta9-tetrahydrocannabinol (THC), the main psychoactive substance in marijuana plant, did not render measurable effects during in vivo and ex vivo testing of cerebral artery/arteriole diameter in adult male and female Sprague–Dawley rats [13], data obtained from human subjects in vivo documented increases in both blood velocity [18, 19] and cerebrovascular resistance [20] following marijuana use. While differences in chemical composition between purified THC and multi-component marijuana leaf may explain the different outcomes, the use of anesthesia in invasive studies with animals introduces an additional variable that needs to be factored in.

Although alcohol consumption concomitant with marijuana has been reported as a trigger of cannabis-related strokes [21], studies on the effects of simultaneous alcohol-marijuana administration on cerebral artery or arteriole diameter are scarce. Our recent studies in male and female rats focused on THC as a major psychoactive substance with one of the highest percentual presence in marijuana leaves [13, 22]. The study by Slayden et al. [13] demonstrated that the in vivo constriction of cerebral arteries by simultaneous alcohol and THC administration in each sex did not statistically differ from constriction produced by alcohol alone.

Ethanol is known to interact with commonly used anesthetics, including general anesthesia, barbiturates and, to a certain degree, morphine [23, 24]. Unlike EtOH, THC interactions with commonly used anesthetics remain largely unknown. Here, we used adult male and female Sprague–Dawley rats to address a potential influence of commonly used anesthetics ketamine/xylazine (KX) and isoflurane on the effect of THC and its combination with alcohol on cerebral arteriole diameter. We also compared this influence with the effect of KX versus isoflurane anesthesia formulations on the degree of EtOH-induced constriction of cerebral arterioles.

## Materials and methods

### Ethical aspects of research

The care of animals and experimental protocols were reviewed and approved by the Institutional Animal Care and Use Ethics Committee of the University of Tennessee Health Science Center, which is an institution accredited by AAALAC international. All animals used were considered to be SPF for common rodent/rat pathogens (free from *Filobacterium rodentium, Clostridium piliforme, Mycoplasma pulmonis, Pneumocystis carinii, Encephalitozoon cuniculi,* Pinworms, fur mites, all rat parvoviruses, rotavirus, Lymphocytic Choriomeningitis Virus, Murine Adenovirus type 1 and 2, Pneumonia virus of mice/rats, Rat polyomavirus 2, Rat Coronavirus / Sialodacryoadenitis Virus, Reovirus 3, Rat theilovirus, and Sendai virus). All methods were performed in accordance with the relevant guidelines and regulations.

### Ethics approval and consent to participate

The study is reported in accordance with ARRIVE guidelines.

### Cranial window in vivo

Male and female adult Sprague–Dawley rats (10–12 weeks of age, Envigo) were weighed and anesthetized with a ketamine/xylazine (KX) mixture (91/9 mg/kg of weight) and kept anesthetized for the duration of the experiment with subsequent ketamine (K) doses (50 mg/kg of weight) every 15 min or as needed. For experiments under isoflurane anesthesia, animals were induced with 5% isoflurane (Kent Scientific Corporation, SomnoSuite, room air) and then maintained on 2.5–4% isoflurane for the duration of surgery and drug testing. Females were subjected to pap smears and used in proestrus, diestrus or metestrus to avoid the possible influence of the estrogen dip, which occurs during estrus. Estrous cycle stage was determined through analysis of vaginal cytology following a previously published approach [25]. Heat support was provided to anesthetized animals using a surgical warming platform (Kent Scientific Corporation, Torrington, CT, USA). A catheter was inserted in the carotid artery to ensure that the infused drugs were directed toward the brain rather than the thoracic cavity. A cranial window was made on the side where the catheter had been inserted. For this purpose, the area above the zygomatic arch between the ear and the eye was cleared of hair, skin and underlying tissue. Then, the bone was removed using a Dremel 4000 instrument (Dremel, Mt. Prospect, USA). The exposed arterioles branching out from the middle cerebral artery were monitored using a Leica MC170 HD microscope with a mounted camera (M125 C; Leica, Buffalo Grove, USA) connected to a computer monitor. Throughout the experiment, the exposed surface of the brain was kept moist with 0.9% NaCl. During surgery, animals did not experience substantial bleeding, cerebrospinal fluid leaks, eye bulging or seizures, and we did not observe any swelling or shrinkage of the brain. Throughout surgery, rectal temperature was documented by readings every 15 min.

For drug infusions, rats were randomly divided into five groups according to the identity of compound that was infused into the cerebral circulation via intracarotid artery catheter. These groups include: 0.9% NaCl, 50 mM EtOH, dimethyl sulfoxide (DMSO)-containing vehicle, 42 nM THC, or 50 mM EtOH combined with 42 nM THC (“simultaneous EtOH with THC” group). Drugs were diluted to their final concentration in sodium saline (0.9% NaCl) and administered via catheter at 1 ml/250 g of weight at a rate 1 mL/min. Each animal was only tested with one drug. Cranial window images before and after drug administration were acquired every 1 min for subsequent analysis.

After effect of drug infusion has been recorded for 15 min, animals were euthanized by intracarotid bolus of ketamine (17 mg/animal at 50 mg/mL) followed by thoracotomy.

### Chemicals

Ethanol (190-proof), THC at 1 mg/mL of methanol, and all other chemicals were purchased from Sigma-Aldrich. THC was prepared as we recently reported [13]. Specifically, before the experiment, methanol was evaporated under the stream of N_2_ gas in the hood, and THC was re-dissolved in either DMSO or EtOH to render 30.18 mM THC stock. THC stock solution and EtOH were diluted into saline (0.9% sodium chloride) to reach final concentrations immediately before experimental use [13].

### Data analysis and statistics

While surgery was performed by one investigator, quantification of arteriole diameter was performed by another person. All rats were number-coded, and person who did not have any influence over data analysis held the key. Analysis was performed as we recently described [13], namely, arteriole diameter measurement was obtained from cranial window images using ImageJ. After diameter measurements were obtained and quantified for each rat under different drug exposures, data were grouped based on previously blinded experimental group identity.

With the exception of data presented in Figures 6-7, statistical analysis was performed using InStat3.05 software (GraphPad, San Diego, CA, USA), as detailed in our recent work [13]. Data from Figures 6-7 were analyzed using IBM SPSS software (Chicago IL, USA). Distribution of data was checked using Kolmogorov–Smirnov approach via built-in function in InStat3.05 software (GraphPad). For non-normal distribution of data, and when the type of distribution could not be established with certainty (number of observations < 10), statistical methods included Mann–Whitney test for two experimental groups and Kruskal–Wallis test with Dunn’s post-test for comparison of three and more experimental groups. For detection of possible interaction between THC and EtOH, two-way analysis of variance was performed using built-in function in Origin 2020. Significance was set at *P* < 0.05, > 80% power. Unless stated otherwise, data are expressed as means ± S.D. In each experimental group, individual diameter recordings in vivo were obtained from different animals. Final plotting and fitting of data were conducted using the Origin 2020.

## Results

### Validation of anesthetic performance

For studies with KX anesthesia, a total of 22 male (389 ± 41 g) and 22 female (286 ± 8 g) rats were used. Isoflurane studies consisted of a total of 28 male (320 ± 16 g) and 20 female (272 ± 14 g) rats. Upon induction with KX mixture (91/9 mg/kg of weight), male rats lost response to a toe-pinch within 15.18 ± 4.82 min. This time did not differ from females. In the female group, lack of toe-pinch response was reached within 16.06 ± 3.6 min. In the group induced with isoflurane (5%), deep anesthesia evident by the lack of toe-pinch response was achieved almost immediately upon isoflurane introduction. Thus, for the isoflurane group, accurate estimation of the time needed to reach deep anesthesia could not be accomplished.

**Fig. 1:**
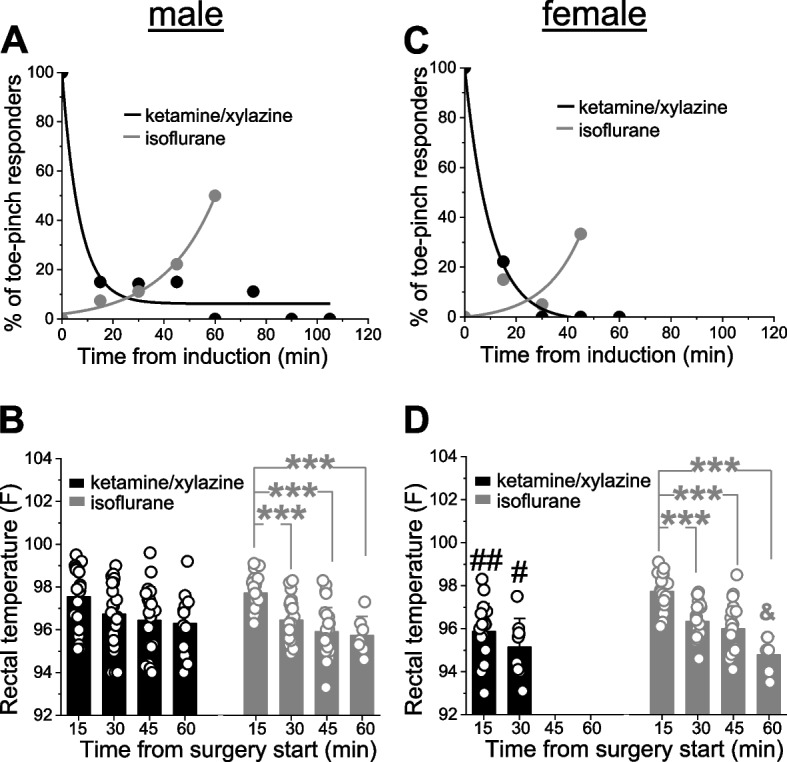
Toe-pinch responses and rectal temperature readings in male and female rats validate KX and isoflurane anesthesia performance. **A** Percent of toe-pinch responding male rats among those which were induced with KX versus isoflurane and maintained with ketamine or isoflurane, respectively, at different time-points during catheter placement and cranial window surgery. If animal responded to a toe-pinch, ketamine or isoflurane within maintenance dose was increased. Here and in C, data were fitted with exponential decay or exponential growth functions of first order using built-in fitting protocol in Origin 2022 (OriginLab Corp). For males under KX, R-square reflecting goodness of fit was 0.96, for males under isoflurane R reached 0.99. **B** Scattered graph showing rectal temperature readings in males under either KX or isoflurane anesthesia at different time-points (in minutes) following induction with KX or isoflurane, respectively. Here and in D, data are shown as mean ± S.D. ***Statistically significant difference, < 0.001 by ANOVA with Tukey post-test. **C** Percent of toe-pinch responding female rats among those which were induced with KX versus isoflurane and maintained with ketamine or isoflurane, respectively, at different time-points during catheter placement and cranial window surgery. If animal responded to a toe-pinch, ketamine or isoflurane within maintenance dose was increased. For females under KX, R-square reflecting goodness of fit was 0.99, for females under isoflurane R reached 0.72. **D** Scattered graph showing rectal temperature readings in females under either KX or isoflurane anesthesia at different time-points (in minutes) following induction with KX mixture or isoflurane, respectively. ##Statistically significant difference compared to the corresponding time-point in males, *p =* 0.000483 by 2-tail t-test; #statistically significant difference compared to the corresponding time-point in males, *p =* 0.012905 by 2-tail t-test. After 30 min, females lacked reply to a toe-pinch under KX (Fig. 1C). Thus, surgeries in females under KX anesthesia were completed faster than in males resulting in missing temperature readings at 30 and 45 min. ***Statistically significant difference, < 0.001 by ANOVA with Tukey post-test. &Statistically significant difference from rectal temperature at 30 and 45 min, *p* < 0.05 by ANOVA with Tukey post-test

After induction with KX mixture, animals were maintained with ketamine (50 mg/kg of weight) and assessed for a toe-pinch response every 15 min. If there was a response to a toe-pinch, additional dose of ketamine was administered until toe-pinch response disappeared and carotid artery catheter placement along with cranial window surgery could be performed. In males, there were approximately 10% of animals that responded to toe-pinch at time-points between 15 and 80 min from anesthesia induction. There were no toe-pinch responses after 80 min under anesthesia (Fig. 1A). Fluctuations in the number of toe-pinch responses in males did not impact rectal temperatures which remain steady; namely, at 0, 15, 30, and 45 min, male rectal temperature readings were: 97.5 ± 1.4, 96.7 ± 1.6, 96.4 ± 1.6, 96.3 ± 1.4 F (Fig. 1B). There were no statistically significant changes in rectal temperature in males over time.

In the isoflurane group, anesthesia was achieved by 2–4% isoflurane as needed to maintain deep anesthesia. The weight of the rat was dialed into the SomnoSuite program to obtain weight-based adjustments of the respiration rate and tidal volume. Notably, males were starting to respond to toe-pinch as more time was passing by from the anesthesia induction (Fig. 1A). If response to a toe-pinch was detected, isoflurane was increased up to 4%. The time-dependent increase in percentage of toe-pinch responders was accompanied by a drop in rectal temperature, this latter drop being consistent with previous literature [26]. Under isoflurane anesthesia, rectal temperatures in males at 0, 15, 30, and 45 min were: 97.7 ± 0.7, 96.4 ± 0.9, 95.9 ± 1.1, 95.1 ± 0.9 F (Fig. 1B). Rectal temperature after 15 min from anesthesia induction in males was significantly higher than at any subsequent time-points (*p* < 0.001 by ANOVA) (Fig. 1B).

Similar to males, the percent of female toe-pinch responses over time dropped in KX group but increased under isoflurane anesthesia (Fig. 1C-D). In females, rectal temperature 15 min after induction with KX was significantly lower than in males, reaching 95.9 ± 1.3 F (*p =* 0.000483 by 2-tail t-test). Rectal temperature in females at 30 min following the induction with KX was also significantly lower than temperatures at the corresponding time-point in males, reaching 95.1 ± 1.3 F (*p =* 0.012905 by 2-tail t-test). Similar to males, however, there was no statistically significant difference in rectal temperatures between time-points. In other words, rectal temperature in females remained stable under ketamine anesthesia following induction with KX (Fig. 1D).

In females under isoflurane anesthesia, rectal temperatures did not differ from rectal temperatures in males at any given time-point. At 0, 15, 30 and 45 min, rectal temperatures in females under isoflurane anesthesia reached 97.7 ± 0.8, 96.3 ± 0.9, 96.0 ± 1.2 and 94.8 ± 0.9, respectively. Similar to males, the rectal temperature 15 min following the induction with isoflurane anesthesia was significantly higher than at any consequent time-point (*p* < 0.001 by ANOVA) (Fig. 1D). Unlike males, however, females exhibited significantly lower rectal temperatures 60 min after induction with isoflurane when compared to either 30 or 45 min (Fig. 1D).

### Anesthetic-specific variability in alcohol action on pial arteriole diameter

**Fig. 2:**
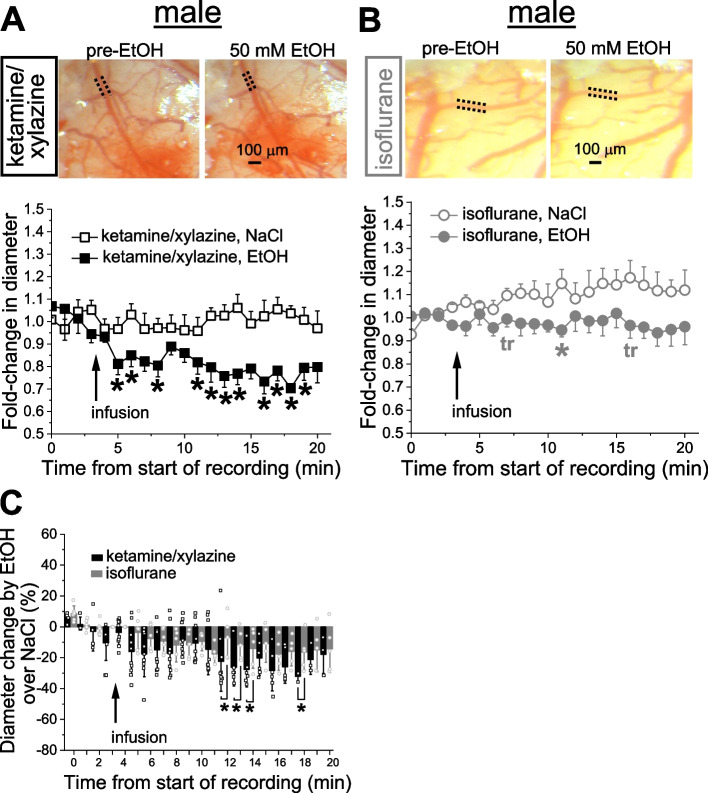
Alcohol effect on pial arteriole diameter under different anesthetics in male rats. **A** Averaged fold-change in male pial arteriole diameter as a function of time following carotid artery infusion of 0.9% NaCl (saline) under KX anesthesia (*n =* 8) or 50 mM EtOH under KX (*n =* 10). Asterisks (*) reflect statistically significant difference between saline and 50 mM EtOH groups with 0.0119 ≤ *p* ≤ 0.046 by 1-tail Mann–Whitney test. For each group here and in Figs. 6B, 7A-B, 2A-B, 3A-B, 4A-B, and 5A-B, data from individual rats were normalized to their respective arteriole diameter at the beginning of diameter monitoring. For visual clarity, in all aforementioned panels, data are presented as mean ± S.E. Here and in B, inserts are of representative cranial window images from male rats showing vasoconstriction by 50 mM EtOH under KX versus isoflurane anesthesia. **B** Averaged fold-change in male pial arteriole diameter as a function of time following carotid artery infusion of 0.9% NaCl under isoflurane (*n =* 6), and 50 mM EtOH under isoflurane (*n =* 6). Asterisk reflects statistically significant difference with *p =* 0.02 by 1-tail Mann–Whitney test. tr: statistical trend with 0.05 ≤ *p* ≤ 0.10 by 1-tail Mann–Whitney test. **C** Scattered graph showing EtOH effect on male pial cerebral arteriole diameter in male rats under ketamine versus isoflurane anesthesia. Each datapoint within each group was normalized to averaged effect of 0.9% NaCl at the same time-point within respective group. Asterisks depict statistically significant differences with 0.0179 ≤ *p* ≤ 0.0411 by 1-tail Mann–Whitney test

 After catheter placement and cranial window surgery were completed, animals were subjected to a body weight-adjusted bolus infusion of 50 mM ethanol (EtOH) into carotid artery toward cerebral circulation. Experiments were performed in a group of animals under KX anesthesia and compared to a separate group of animals under isoflurane anesthesia. For each type of anesthetic, control groups were receiving infusions of 0.9% sodium saline (NaCl). Under KX anesthesia, male cerebral arteriole constricted in a time-dependent manner upon infusion of EtOH when compared to infusion of sodium saline (Fig. 2A).

The drop in arteriole diameter reached statistically significant level as early as 8 min following EtOH infusion and constituted at this point 18.1 ± 15.8% (*p =* 0.046 by 1-tail Mann–Whitney test) from the baseline diameter averaged over 4 min pre-infusion (Fig. 2A). Under isoflurane anesthesia, however, the EtOH-induced constriction in males was mild (Fig. 2B). When normalized to time-matched effect of sodium saline, EtOH-induced constriction under isoflurane anesthesia was significantly smaller at several time-points during observation when compared to the same time-points under KX anesthesia (Fig. 2C).

**Fig. 3:**
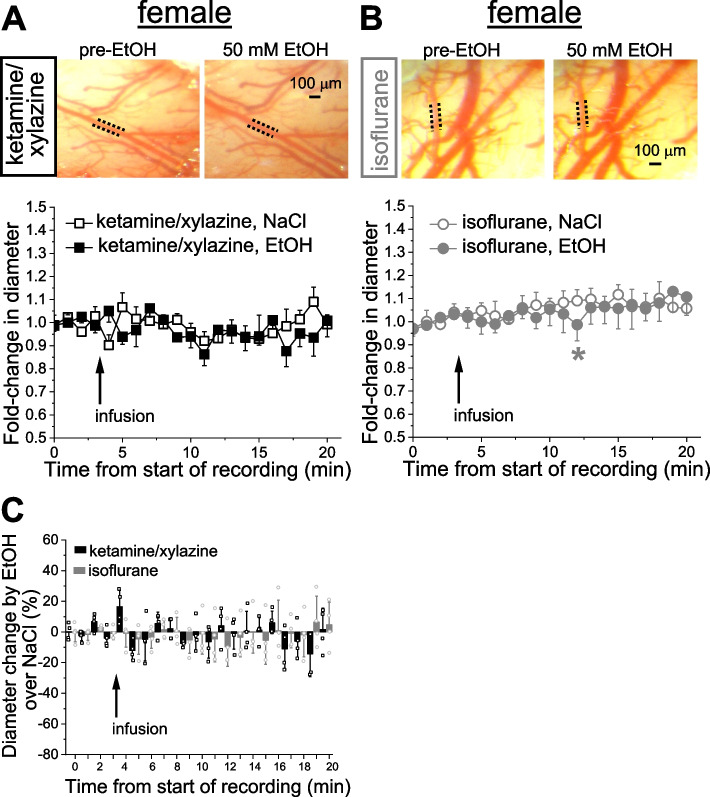
Alcohol effect on pial arteriole diameter under different anesthetics in female rats. **A** Averaged fold-change in female pial arteriole diameter as a function of time following carotid artery infusion of 0.9% NaCl (saline) under KX anesthesia (*n =* 4) or 50 mM EtOH under KX (*n =* 4). Here and in B, representative cranial window images from female rats showing lack of vasoconstriction by 50 mM EtOH disregard the type of anesthetic used. **B** Averaged fold-change in female pial arteriole diameter as a function of time following carotid artery infusion of 0.9% NaCl under isoflurane (*n =* 4), and 50 mM EtOH under isoflurane (*n =* 4). **C** Scattered graph showing lack of EtOH-induced constriction in female pial cerebral arterioles under KX or isoflurane anesthesia. Each datapoint within each group was normalized to averaged effect of 0.9% NaCl at the same time-point within respective group

In female rats under KX anesthesia, we have recently reported significantly smaller EtOH-induced constriction of pial arterioles than in males [13]. Indeed, in our present study EtOH-induced constriction under KX anesthesia was largely absent (Fig. 3A). Ethanol action on cerebral arteriole diameter was also absent when female rats were anesthetized using isoflurane anesthesia (Fig. 3B-C) indicating a strong sex differential to constriction caused by EtOH.

### Anesthetic-specific variability in THC action on pial arteriole diameter

**Fig. 4:**
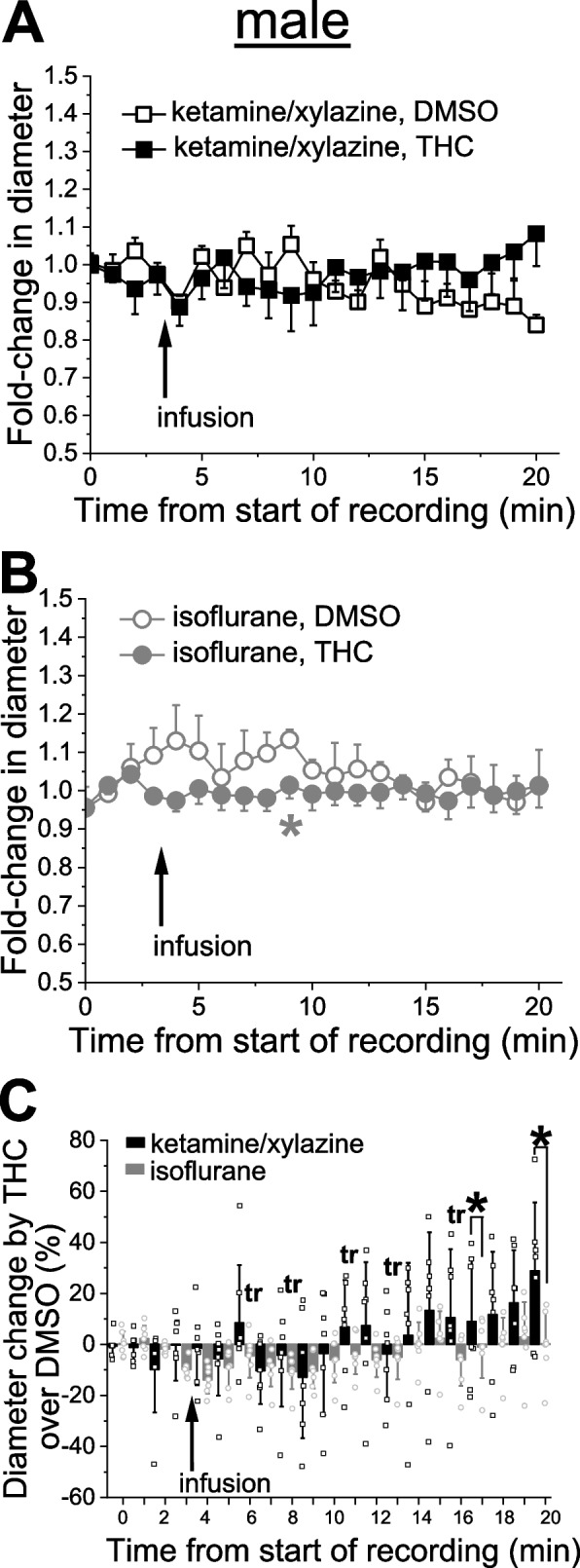
THC effect on pial arteriole diameter in male rats is influenced by anesthetics. **A** Averaged fold-change in male pial arteriole diameter as a function of time following carotid artery infusion of DMSO-containing saline under KX anesthesia (*n =* 4) or 42 nM THC under KX (*n =* 7). **B** Averaged fold-change in male pial arteriole diameter as a function of time following carotid artery infusion of DMSO-containing saline under isoflurane (*n =* 5), and 42 nM THC under isoflurane (*n =* 6). Asterisk depicts statistically significant difference in isoflurane group with *p =* 0.0026 by 1-tail Mann–Whitney test. **C** Scattered graph comparing THC effect on male pial cerebral arteriole diameter under KX versus isoflurane anesthesia. Each datapoint within each group was normalized to averaged effect of DMSO-containing saline at the same time-point within respective group. Asterisks depict statistically significant differences with *p =* 0.0364 by 1-tail Mann–Whitney test. tr: statistical trend with 0.05 ≤ *p* ≤ 0.10 by 1-tail Mann–Whitney test

Probing of THC was conducted on separate animals from the EtOH groups. After catheter placement and cranial window surgery, animals were subjected to a body weight-adjusted bolus infusion of THC stock in DMSO vehicle which was diluted in sodium saline to reach 42 nM THC. Experiments were performed in a group of animals under KX anesthesia and compared to a separate group of animals under isoflurane anesthesia. For each type of anesthetic, control groups were receiving infusions of DMSO. Under KX anesthesia, male cerebral arteriole largely failed to respond with changes in diameter when compared to the infusion of DMSO-containing saline (Fig. 4A). Under isoflurane anesthesia, however, the THC tended to constrict pial arterioles, reaching statistically significant constriction at 6 min following the infusion. At this time-point, THC constriction reached 10.4 ± 6.8% (*p =* 0.026 by 1-tail Mann–Whitney test) when compared to corresponding time-point with DMSO-containing saline (Fig. 4B). When normalized to time-matched effect of DMSO-containing saline, however, THC effect under isoflurane anesthesia was significantly smaller at several time-points during observation when compared to the same time-points under KX (Fig. 4C).

**Fig. 5:**
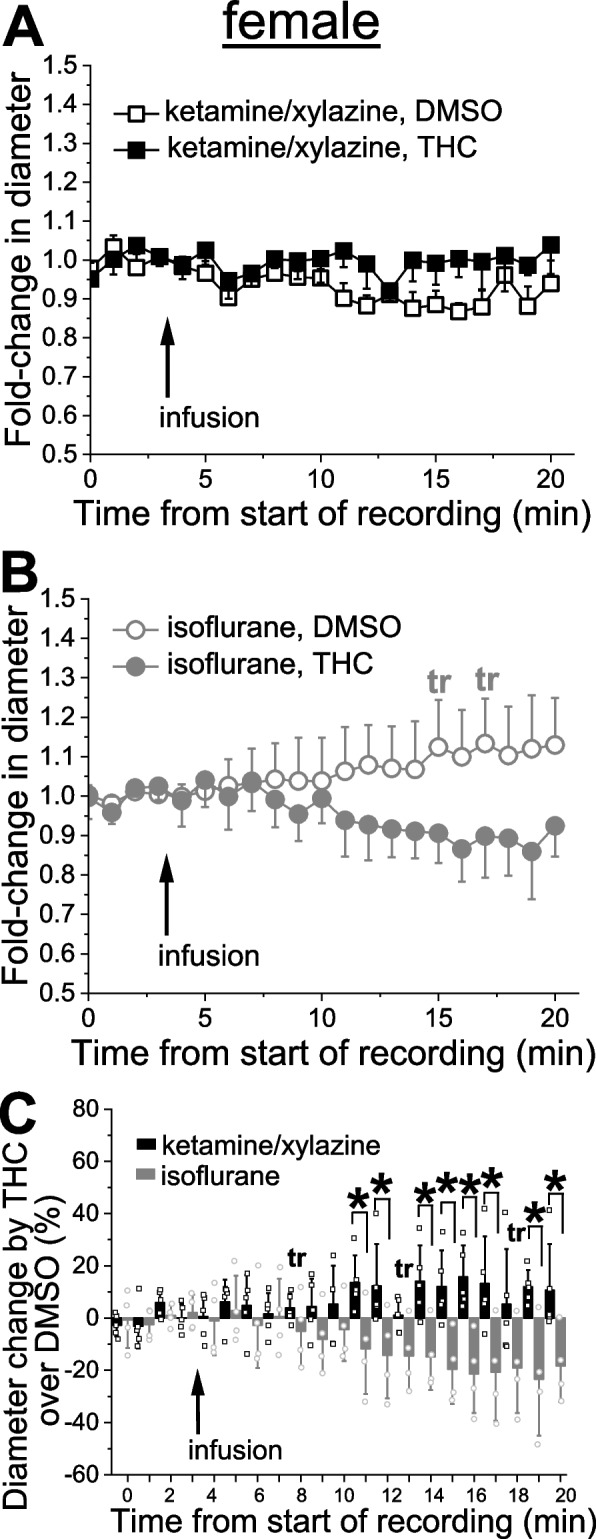
THC effect on pial arteriole diameter in female rats is influenced by anesthetics. **A** Averaged fold-change in female pial arteriole diameter as a function of time following carotid artery infusion of DMSO-containing saline under KX anesthesia (*n =* 4) or 42 nM THC under KX (*n =* 5). **B** Averaged fold-change in female pial arteriole diameter as a function of time following carotid artery infusion of DMSO-containing saline under isoflurane (*n =* 4), and 42 nM THC under isoflurane (*n =* 4). Here and in C, tr: statistical trend with 0.05 ≤ *p* ≤ 0.10 by 1-tail Mann–Whitney test. **C** Scattered graph comparing THC effect on female pial cerebral arteriole diameter under KX versus isoflurane anesthesia. Each datapoint within each group was normalized to averaged effect of DMSO-containing saline at the same time-point within respective group. Asterisks depict statistically significant differences with 0.0079 ≤ *p* ≤ 0.0317 by 1-tail Mann–Whitney test

Similar to males, female rats under KX anesthesia exhibited little sensitivity to THC (Fig. 5A) [13]. Although pial arteriole diameter tended to decrease upon THC infusion under isoflurane anesthesia when compared to DMSO-containing saline, due to high variability and thus, standard error of readings, only statistical trends (*p* ≤ 0.1) were reached (Fig. 5B). When normalized to time-matched effect of DMSO-containing saline, THC effect under isoflurane anesthesia was significantly different at numerous time-points during observation when compared to the same time-points under KX (Fig. 5C).

### Anesthetic effect on simultaneous administration of alcohol and THC

**Fig. 6:**
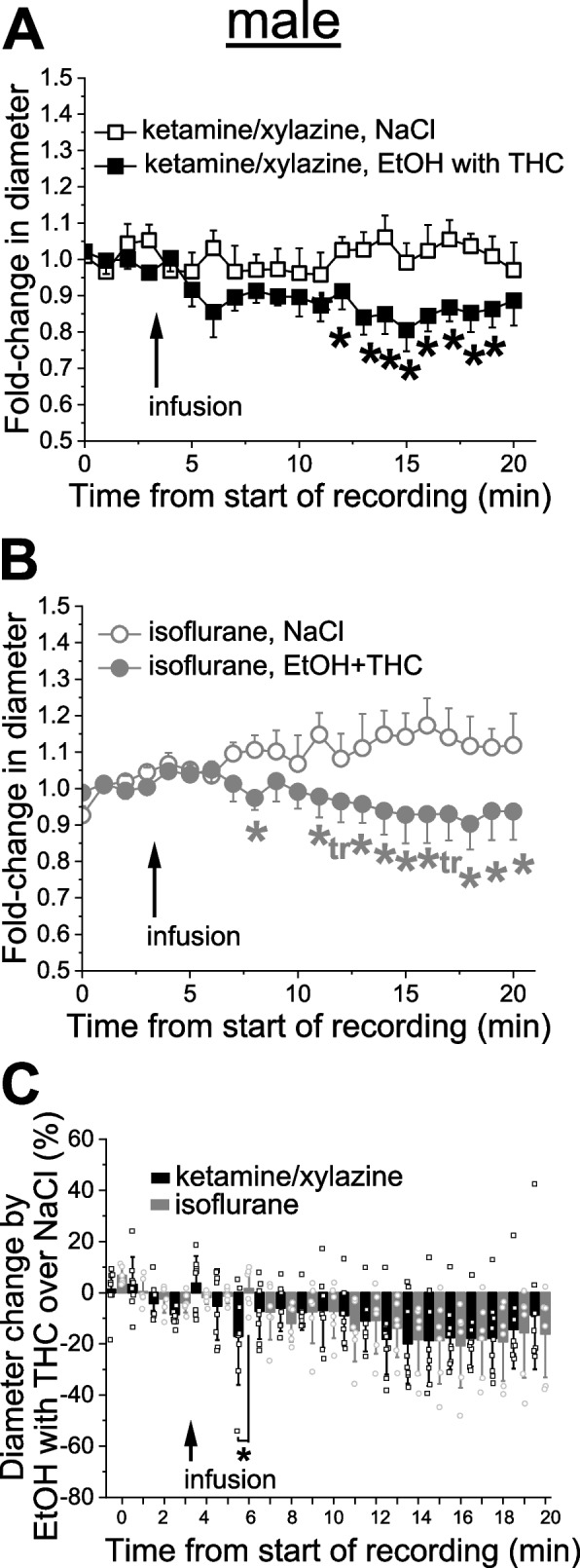
Anesthetics largely fail to alter effect of simultaneous application of alcohol with THC on cerebral arteriole diameter in male rats. **A** Averaged fold-change in male pial arteriole diameter as a function of time following carotid artery infusion of 0.9% NaCl (saline) under KX anesthesia (*n =* 8) or 50 mM EtOH with 42 nM THC under KX (*n =* 11). Asterisks (*) reflect statistically significant difference between saline and 50 mM EtOH with 42 nM THC groups with 0.025 ≤ *p* ≤ 0.031 by 1-tail Mann–Whitney test. **B** Averaged fold-change in male pial arteriole diameter as a function of time following carotid artery infusion of 0.9% NaCl under isoflurane (*n =* 6), and 50 mM EtOH with 42 nM THC under isoflurane (*n =* 6). Asterisks (*) reflect statistically significant difference between saline and 50 mM EtOH with 42 nM THC groups with 0.013 ≤ *p* ≤ 0.0465 by 1-tail Mann–Whitney test. tr: statistical trend with 0.05 ≤ *p* ≤ 0.10 by 1-tail Mann–Whitney test. **C** Scattered graph showing effect of simultaneous application of 50 mM EtOH with 42 nM THC on male pial cerebral arteriole diameter in male rats under ketamine versus isoflurane anesthesia. Each datapoint within each group was normalized to averaged effect of 0.9% NaCl at the same time-point within respective group. Asterisk depicts statistically significant differences with *p =* 0.0145 by 1-tail Mann–Whitney test

Animals were subjected to simultaneous infusion of 50 mM EtOH and 42 nM THC. This mixture was free of DMSO as EtOH served as a vehicle for THC. Under KX anesthesia, male cerebral arteriole constricted in a time-dependent manner upon infusion of EtOH and THC when compared to infusion of sodium saline (Fig. 6A). The drop in arteriole diameter reached statistically significant level at 9 min following infusion and constituted at this point 20.0 ± 15.6% (*p =* 0.0075 by 1-tail Mann–Whitney test) from the baseline diameter averaged over 4 min pre-infusion (Fig. 6A). Constriction was also observed under isoflurane anesthesia. The first drop in arteriole diameter reached statistically significant level at 9 min following infusion and constituted at this point 11.9 ± 7.3% constriction over pre-drug values from baseline diameter (*p =* 0.0205 by 1-tail Mann–Whitney test) from the baseline diameter averaged over 4 min pre-infusion (Fig. 6B). When normalized to time-matched effect of sodium saline, constriction by simultaneous infusion of EtOH with THC under isoflurane anesthesia was significantly smaller only at a single time-point during the whole period of monitoring, when compared to the same time-points under KX anesthesia (Fig. 6C).

**Fig. 7:**
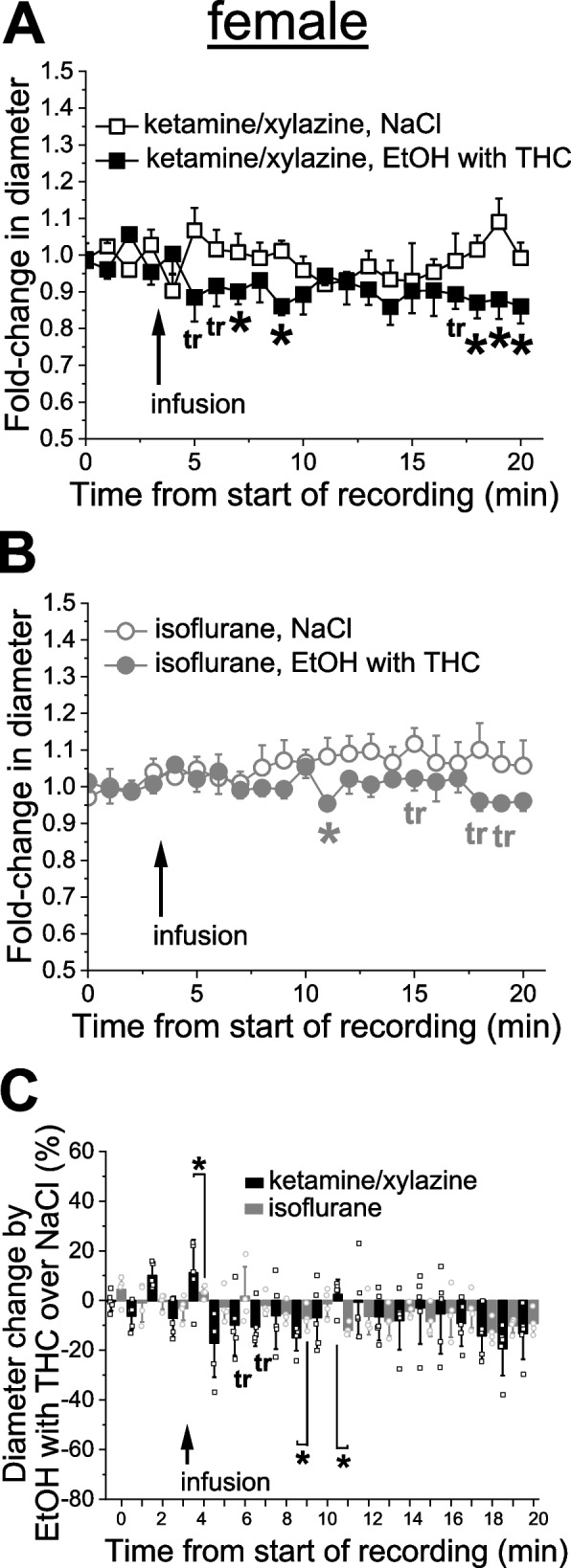
Effect of simultaneous administration of alcohol with THC on pial arteriole diameter under different anesthetics in female rats. **A** Averaged fold-change in female pial arteriole diameter as a function of time following carotid artery infusion of 0.9% NaCl (saline) under KX anesthesia (*n =* 4) or 50 mM EtOH with 42 nM THC under KX (*n =* 5). Asterisks (*) reflect statistically significant difference between saline and 50 mM EtOH with 42 nM THC groups with 0.021 ≤ *p* ≤ 0.0317 by 1-tail Mann–Whitney test. tr: statistical trend with 0.05 ≤ *p* ≤ 0.10 by 1-tail Mann–Whitney test. **B** Averaged fold-change in female pial arteriole diameter as a function of time following carotid artery infusion of 0.9% NaCl under isoflurane (*n =* 4), and 50 mM EtOH with 42 nM THC under isoflurane (*n =* 4). Asterisk depicts statistically significant differences with *p =* 0.0285 by 1-tail Mann–Whitney test. tr: statistical trend with 0.05 ≤ *p* ≤ 0.10 by 1-tail Mann–Whitney test. **C** Scattered graph showing effect of simultaneous application of 50 mM EtOH with 42 nM THC on male pial cerebral arteriole diameter in female rats under ketamine versus isoflurane anesthesia. Each datapoint within each group was normalized to averaged effect of 0.9% NaCl at the same time-point within respective group. Asterisks (*) reflect statistically significant difference between isoflurane and ketamine groups with 0.021 ≤ *p* ≤ 0.0317 by 1-tail Mann–Whitney test. tr: statistical trend with 0.008 ≤ *p* ≤ 0.0365 by 1-tail Mann–Whitney test

In female rats under KX anesthesia, cerebral arteriole also constricted in a time-dependent manner upon infusion of EtOH with THC when compared to infusion of sodium saline (Fig. 7A). The drop in arteriole diameter reached statistically significant level as early as 4 min following infusion and constituted at this point 10.6 ± 7.8% constriction over pre-drug diameter (*p =* 0.0286 by 1-tail Mann–Whitney test) from the baseline diameter averaged over 4 min pre-infusion (Fig. 7A). Under isoflurane anesthesia, statistically significance constriction was only observed 8 min following infusion and constituted at this point 11.8 ± 2.4% (*p =* 0.0285 by 1-tail Mann–Whitney test) from the baseline diameter averaged over 4 min pre-infusion (Fig. 7B). When normalized to the time-matched effect of sodium saline, constriction by simultaneous infusion of EtOH and THC under isoflurane anesthesia was significantly smaller at several time-points during observation when compared to the same time-points under KX anesthesia (Fig. 7C). Yet, at one time-point (8 min post-infusion), constriction under isoflurane anesthesia significantly exceeded the constriction evoked by administration of EtOH and THC under KX (*p =* 0.008 by1-tail Mann–Whitney test).

## Discussion

In the present study, we compared the influence of two commonly used anesthetics, injectable KX and inhalant (gaseous) isoflurane, over EtOH and THC actions on pial arteriole diameter in Sprague–Dawley rats. Regarded as a “multifaceted drug”, ketamine is widely used in clinical practice [27] and bench studies with laboratory animals [28, 29]. Ketamine analgesic, neuroprotective, anti-inflammatory, and antitumor effects have been reported [29, 30]. However, ketamine use in EtOH-affected populations is limited due to pro-convulsive properties [24]. As for isoflurane, it is a popular choice of inhalational anesthesia owing to its lack of pro-convulsive effects [24]. Moreover, an increasing number of reports point at the therapeutic properties of isoflurane regimens. For instance, isoflurane was found to decrease cocaine- and nicotine-reinforced responses in rats when compared to isoflurane-free littermates [31]. In addition, brief isoflurane administration protocol ameliorated motor deficits in a rat model of early-stage Parkinson’s disease [32]. Low-levels of isoflurane (0.75% and 1.5%) demonstrated increasing cardioprotective properties against stress-induced cardiomyopathy in rat study [33]. Also in a rat model, isoflurane was proven superior to subcutaneous fentanyl/fluanisone/midazolam in supporting resuscitation efforts following cardiac arrest [34].

The advantageous consideration of isoflurane anesthesia based on our current study comes from the finding that deep anesthesia measured by the lack of the toe-pinch response was achieved almost immediately, allowing rapid surgical tolerance. Unexpectedly, we also observed several toe-pinch responses at later time-points under isoflurane. This could be due to a sensation-accumulation effect, leading to the need for further anesthetics as was done in our work (increase from 2 or 2.5% to 4% isoflurane as surgeries were progressing). A possible further study could include administration of preoperative analgesics, which are known to lead to more constant/consistent anesthesia. In contrast to instantaneous effect of isoflurane inhalation, ketamine anesthesia following induction with KX injection required on average 15–16 min to achieve lack of toe-pinch response. Performance of KX versus isoflurane anesthetics similar to our findings has been reported for other laboratory species, i.e., male and female rice rats *Oryzomys palustris* [35]. However, while isoflurane in our study led to a decline in rectal temperature in both males and females (Fig. 1B, D), this anesthetic failed to do so in rice rats placed on a heated platform and wrapped with multipurpose sealing wrap (Glad Press’n Seal) during a tooth extraction procedure [35]. This discrepancy in anesthetic performance may arise from species-based variability in the effect of anesthesia on physiological parameters. It may also be due to the absence of a cling-film draping material in our studies, which have been shown previously to prevent hypothermia in other rodent species [36]. Further studies would be needed to confirm either hypothesis.

Isoflurane and KX also exhibit differential influences over physiological and pharmacological responses. For example, comparison of auditory brainstem responses in young adult male Long-Evans rats revealed higher hearing thresholds under isoflurane anesthesia when compared to KX [37]. In adult male Wistar rats, KX anesthesia led to a global reduction in brain glucose metabolism measured by ^18^F-fluorodeoxyglucose-positron emission tomography compared to isoflurane [38]. In a study with adult male Sprague–Dawley rats, isoflurane decreased respiratory rate while deep anesthesia with ketamine increased it [39]. Interestingly, such differential actions were reported even though both anesthetics decreased ventilatory pattern variability [39]. Moreover, both ketamine and isoflurane were reported to exert a depressing effect on cerebral interstitial oxygen saturation (pO_2_) as measured by low frequency electron paramagnetic resonance spectroscopy with lithium phthalocyanine in male Wistar rats (200–250 g) [40]. KX combinations have been shown to cause hypotension, hypoventilation, decreased depth of ventilation, and apnea [41–43].

Within cardiovascular system, KX and isoflurane are known to exert a differential influence on systemic blood pressure and heart rate [44]. Choice of anesthesia also affects outcomes when pathological conditions are modeled at the bench setting. For example, in a rat model of hemorrhagic/traumatic shock induced by midline laparotomy and bleeding with subsequent resuscitation under several types of anesthetics, histological analysis of several organs pointed at shock-induced damage under KX but not in the isoflurane group [45]. In male Wistar rats, a smaller mortality following middle cerebral artery occlusion was reported for isoflurane anesthesia when compared with KX [46]. Yet, both KX and isoflurane without oxygen supplementation cause hypoxia, hence, lead to vasoconstriction [41, 42]. In synthesis, there is abundant experimental evidence suggesting the existence of differential as well as common body/system targets for isoflurane and KX.

In our work, isoflurane but not KX produced a time-dependent drop in rectal temperature in both male and female rats (Fig. 1B and D). This observation is in line with previous reports, as isoflurane is known for its hypothermic properties in non-hibernating species such as rat [26]. Hypothermia under isoflurane anesthesia observed in our present work could not be explained by a greater depth of anesthesia, as while rectal temperatures were dropping, the percent of toe-pinch responders was increasing in both sexes (Fig. 1). Thus, the likely explanation resides in a reported effect of isoflurane on the thermoregulatory center in preoptic area of hypothalamus. Here, microdialysis study on adult male Wistar rats documented isoflurane-driven increase in norepinephrine release while norepinephrine levels in the posterior hypothalamus remained unchanged [47]. Moreover, systemic effects of isoflurane in cardiovascular system might also contribute to isoflurane-characteristic hypothermia. Detailed study on nine weeks-old male Sprague–Dawley rats reported steady, time-dependent decrease in systolic blood pressure and heart rate under isoflurane, but not KX anesthesia [44]. Isoflurane-driven hypothermia, however, remains an unwanted feature during prolonged surgical procedures. Further studies could investigate whether additional methods of heat support, such as pre-warming or draping with a sterile cling-drape would prevent the drop in temperature that is characteristic of isoflurane.

One of the major findings in our work is that in male rats, EtOH-induced constriction of pial arterioles was smaller under isoflurane anesthesia when compared to KX (Fig. 2C). The exact mechanism(s) that enables influence of anesthetics used in present study over alcohol vasoactive property in cerebral circulation remains elusive. Potential mechanisms may include canonical pathways of drug-drug interactions. For instance, EtOH is known to inhibit drug metabolism; thus, less general anesthetic is needed in individuals who chronically abuse EtOH or subjects under acute EtOH intoxication (reviewed by 24). Whether there is any influence of anesthesia over EtOH metabolism, remains to be determined. However, this is unlikely, as if this were the case, then we would observe similar constriction of cerebral arteries at the initial time-points of EtOH infusion under KX or isoflurane anesthesia. This similarity would diminish as EtOH metabolization is affected by either of anesthetics. Yet, this was not the case, as differential constriction was observed almost immediately between KX and isoflurane groups (Fig. 2A-B). An intriguing explanation may arise from microspectrophotometry work in the anterior cortex, posterior cortex, and pons of male Long Evans rats (350–450 g): these animals show no changes in the naturally present heterogeneity of oxygen saturation levels within small cerebral veins in response to ketamine [48]. In contrast, isoflurane was reported to decrease such heterogeneity [49]. Thus, anesthetics may differentially alter coupling between regional cerebral blood flow and brain tissue oxygenation. Should EtOH have vascular, and neuronal/glial targets, dismantling of their coordination would lead to differential vasoconstriction under various anesthetics. Finally, anesthetics might be interfering with EtOH-sensing site(s) within cerebral vasculature itself. Ethanol-induced constriction of cerebral arteries has been shown to be mediated via calcium-/voltage-gated potassium channels of large conductance (BK) in the vascular smooth muscle [14, 50]. Additional molecular targets for cerebrovascular effect of EtOH include ryanodine receptors [51], and transient receptor potential cation channel subfamily V member 1 [52]. Orthosteric or allosteric modulation of these receptors by KX and isoflurane resulting in altered EtOH-sensing cannot be ruled out. The precedent for isoflurane interference with ligand-gated channel exists, as isoflurane was shown to increase agonist affinity of the nicotinic acetylcholine receptor via diminished dissociation of the agonist [53].

With regards to THC, we found virtually no effect of this substance on pial arteriole diameter at the concentration tested under KX, and a slight constriction under isoflurane in both males and females (Figs. 4 and 5). In both sexes, comparison of THC effect under KX versus isoflurane rendered statistically significant difference (Figs. 4C, 5C). This observation is important while comparing results from different laboratories utilizing different anesthesia protocols, as change in anesthetic certainly contributed to data variability, as was recognized previously [33, 45, 46].

Compared with EtOH or THC alone, EtOH and THC simultaneous administration resulted in least variability of drug effect on cerebral arteriole diameter whether anesthetic type or sex were considered (Figs. 6 and 7). In males, simultaneous administration of EtOH and THC resulted in robust constriction of cerebral arterioles whether under KX or isoflurane anesthesia (Fig. 6). Albeit mild and less persistent, constriction was also observed in females (Fig. 7A-B). In females, anesthetic type only drove significant differences in the magnitude of constriction at three time-points during observation (Fig. 7C). These observations on the limited influence of anesthetic on the combination of EtOH and THC, as opposed to individual drugs, is consistent with previous reports highlighting the distinct effects of alcohol and marijuana products co-use on the brain [54, 55]. Inability of anesthetics to influence arteriole constriction by combination of alcohol and THC indicates that such combination may interfere with anesthetics. The mode of interference remains speculative at this point but may involve alcohol and THC affecting anesthetics’ relevant molecular targets and/or having new sites of action that negate anesthetic drugs.

Whether our findings from arterioles stemming from middle cerebral artery are widely applicable to cerebral vessels in other brain regions and irrigation territories remains unknown. However, the influence of anesthesia on pharmacology of cerebrovascular tree is likely region-specific. Indeed, ketamine effects on brain glucose uptake/metabolism are heterogenous, depending on the brain region involved [38, 56]. Considering that glucose uptake by the brain is proportional to cerebral blood flow [57, 58], the region-specific depressive effect of ketamine raises question whether ketamine affects cerebrovascular function in heterogenous manner. Such region-specific effect on cerebral blood flow in a forebrain region of adult male Sprague–Dawley rats was detected for ketamine-xylazine by the means of nuclear magnetic resonance perfusion imaging and electron paramagnetic resonance oximetry [59]. Isoflurane effects on the brain are also region-specific. In 3 months-old male Fischer rats, regional variability of isoflurane (1.5%) anesthesia on local glucose utilization has been reported with increased glucose utilization by autoradiographic detection in substantia nigra pars compacta and several structures of limbic systems with concomitant decrease of glucose utilization in cerebellum, red nucleus, and ventral thalamus [60]. Thus, formal testing is needed to determine applicability of our present findings to other brain territories. While performing such testing, additional consideration should be given to age of subjects under study as cerebral artery function and pharmacological characteristics undergo age-dependent transformations that may influence experimental outcomes [61].

### Summary and conclusions

We confirmed previously reported sexual dimorphism in EtOH-induced constriction of cerebral arteries. Moreover, while arteries in males constricted in presence of either one of the anesthetics, type of anesthesia affected degree of such constriction. Regarding THC, arteries from both males and females tended to constrict when THC was tested under isoflurane, this constriction was significantly larger compared to effect under KX anesthesia. Lastly, simultaneous application of alcohol and THC constricted arteries in both males and females with limited influence from the type of anesthetic used. Present findings are critical for bench studies when various anesthetics are used in rodents to model prevalent human pathologies. However, our work extends its relevance to clinical scenarios when subjects who chronically misuse EtOH or are under acute EtOH intoxication have to undergo anesthesia. While anesthesia of patients under influence of recreational drugs (such as EtOH and marijuana) should be generally avoided, it is not always possible. Emergency interventions may require anesthesia administration to intoxicated patient as probability of vehicular accidents is increased by EtOH or marijuana intoxication [62–64]. Although our sequence of administration does not follow clinical setting (anesthesia first, then drug), it provides premise for future studies addressing this sequence.

## Data Availability

The datasets used and/or analyzed during the current study are available from the corresponding author on reasonable request.

## Abbreviations

BK: Calcium- and voltage-gated potassium channels of large conductance
DMSO: Dimethyl sulfoxide
KX: Ketamine/xylazine
THC: (-)-Trans-delta9-tetrahydrocannabinol
